# Case Report of Synchronous Prostate, Hepatocellular, and Rectal Carcinomas and Review of the Literature

**DOI:** 10.1155/2020/6967428

**Published:** 2020-01-21

**Authors:** Viktoria Lamprou, Daniel Paramythiotis, Dimitrios Giakoustidis, Anestis Karakatsanis, Athanasios Astreinidis, Moysis Moysidis, Antonios Mihalopoulos, Stefanos Finitsis

**Affiliations:** ^1^Department of Interventional Radiology, University General Hospital of Thessaloniki AHEPA, Thessaloniki 546 21, Greece; ^2^1st Propaedeutic Surgery Department, University General Hospital of Thessaloniki AHEPA, Thessaloniki 546 21, Greece; ^3^1st Surgery Department, Aristotle University of Thessaloniki, General Hospital Papageorgiou, Thessaloniki 56403, Greece

## Abstract

Synchronous occurrence of three histopathologically distinct malignant tumors is a rare event, and there are no definitive guidelines about the optimal treatment of these patients. We report a case of synchronous prostate, hepatocellular, and rectal carcinomas and discuss our therapeutic strategy that resulted in excellent clinical results.

## 1. Introduction

Synchronous malignancies are defined as tumors diagnosed within two to six months of the initial diagnosis of a primary tumor and occur in 0,002% to 1,96% of cancer patients [1, 2]. This frequency is increasing despite a general decline in cancer deaths since 1991 because of the increased lifespan of the general population secondary to proper screening, earlier diagnosis, improvements in treatment, and surveillance of patients [3]. Management of synchronous cancers is challenging [4]. We present a patient with synchronous liver, colorectal, and prostate cancers treated by a multidisciplinary approach with an excellent clinical outcome.

## 2. Case Presentation

**Figure 1:**
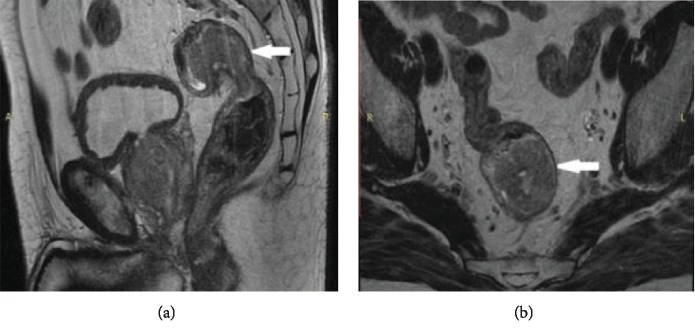
Sagittal (a) and axial (b) reconstruction of a T2-weighted MRI showing the rectal tumor (arrow).

**Figure 2:**
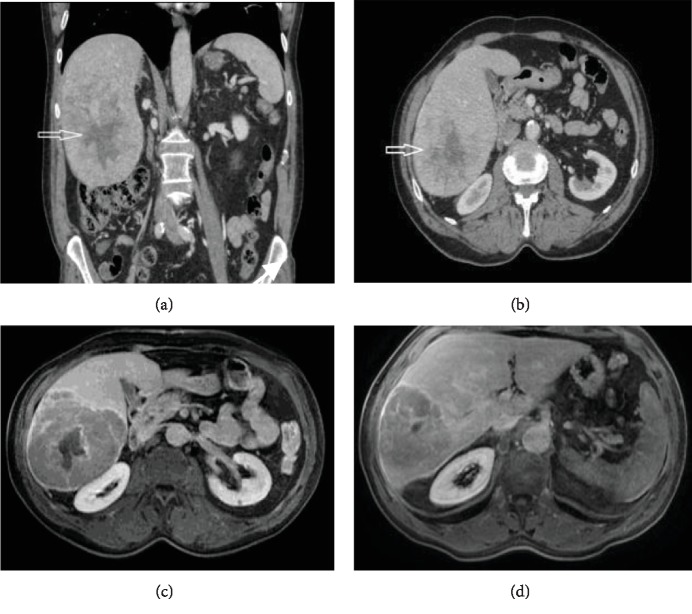
Coronal (a) and axial (b) CT scan images showing a large (>10 cm) inhomogeneously enhancing liver mass with a central scar occupying liver segments V, VI, and VII (arrow). (c, d) Axial post contrast MRI image demonstrating heterogeneously enhancing liver mass with washout and central nonenhancing component, features mostly consistent with atypical HCC.

A 67-year-old man presented with a history of change of bowel habits, blood in stool, and weight loss. His past medical history included only tobacco and alcohol use. According to his family history, two of his first-degree relatives had developed colonic cancer. A colonoscopy revealed the presence of a 4 cm mass approximately 10 cm from the anal verge and was confirmed to be a well-differentiated colon adenocarcinoma at a biopsy. Laboratory tests showed elevated serum carcinoembryonic antigen (CEA) (8,07 ng/ml), elevated serum prostate-specific antigen (PSA) (17,87 ng/ml), and elevated *α*-FP (17,9 ng/ml). The patient was seronegative for HBsAg and anti-HCV (IgM and IgG). His liver function tests were within normal limits. The abdominal MRI showed the presence of an 11 × 10, 5 × 10, 5 cm mass in the right hepatic lobe, with radiologic features consistent with a primary liver lesion (Figures 1 and 2). The CT-guided liver biopsy revealed a well-differentiated hepatocellular carcinoma. Additionally, because of the elevated PSA values, a prostate biopsy was performed. Seven core biopsies were taken from the left prostatic lobe and eight from the right lobe, and all of them were positive for prostatic adenocarcinoma (Gleason score 3 + 4 = 7/10).

**Figure 3:**
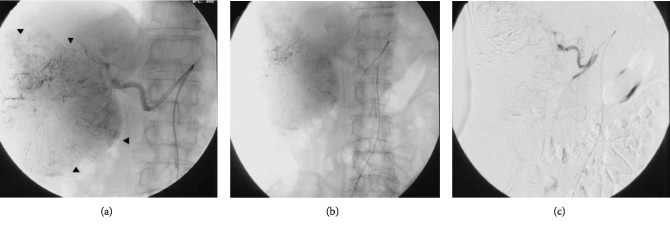
(a) Hepatic arteriogram shows a large tumor staining in the right hepatic lobe (arrowheads). (b, c) Arteriogram after chemoembolization of the tumor, and its feeding branch shows no residual tumor enhancement.

**Figure 4:**
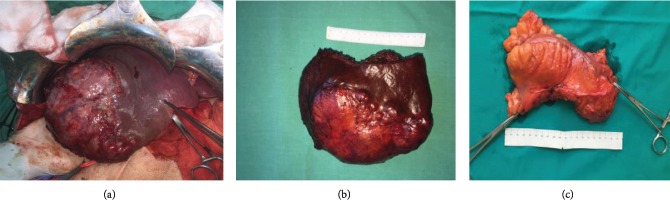
Intraoperative photo of HCC (a). Surgical specimen of the liver (b) and the rectosigmoid colon (c).

**Figure 5:**
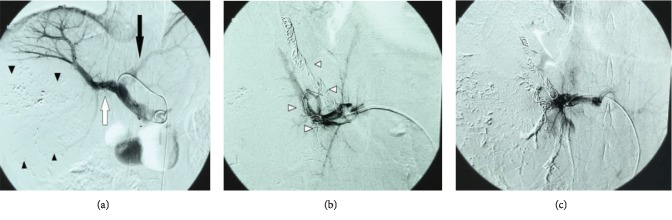
(a) Portal venogram showing the right portal vein (white arrow), the left portal vein (black arrow), and the HCC (black arrowheads). (b) Coils in the anterior and posterior branches of the right portal vein (white arrowheads). (c) Final portal venogram shows occlusion of the right portal vein branches.

**Figure 6:**
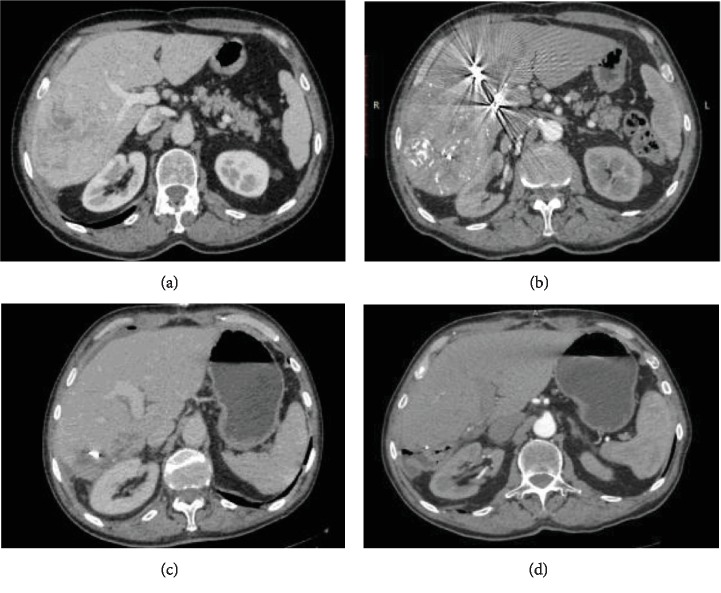
(a) CT scan before TACE and PVE. (b) One month after PVE and three months after TACE, anticipated hypertrophy of LLL is observed. On CT scan, one month (c) and three months (d) after liver resection, there is no evidence of HCC recurrence. Significant hypertrophy of the liver remnant is noted.

Given the patient's good performance status, surgical resection for the hepatocellular carcinoma was considered. However, because of the small liver remnant, it was decided to first perform transarterial chemoembolization, to partially control the hepatocellular carcinoma (Figure 3). Two weeks later, the rectal carcinoma was treated by low anterior resection (Figure 4). After six weeks, right portal vein embolization was performed to allow for hypertrophy of the liver remnant (Figure 5). Finally, one month later, the hepatocellular carcinoma was treated by right hepatectomy (Figures 4 and 6). Active surveillance was adopted for the prostate cancer.

Histopathological evaluation of the rectal specimen showed a moderately differentiated adenocarcinoma Stage IIA (T3N0M0) and a well-differentiated hepatocellular adenocarcinoma Stage IIIA (T3N0M0). Recovery was uneventful, and the patient remained disease-free up until the 2-year follow-up when he died of acute myocardial infarction.

## 3. Discussion

According to the Surveillance Epidemiology and End Results (SEER) project and the International Association of Cancer Registries and International Agency for Research on Cancer (IACR/IARC), synchronous multiple primaries are defined as two or more independent primaries diagnosed within two to six months of the initial diagnosis [4, 5]. Factors associated with increased risk of developing multiple primary malignancies include environmental exposures, genetic factors (family cancer syndromes and other genetic susceptibility factors), lifestyle (tobacco and alcohol consumption), immunodeficiency syndromes (either acquired or inherited), and carcinogenic effects of cancer treatment [6].

Male genital cancers and urinary tract cancers are the most widespread combination of synchronous multiple malignancies, followed by colorectal and noncolorectal digestive tract cancers [2]. Specifically, a synchronous occurrence of rectal and prostate cancers has been described approximately in 0,1%-30,5% of cancer patients [7, 8]. In HCC patients, prostate and rectal tumors coexist in 0,05% and 0,4% of cases, respectively [9, 10]. Triple synchronous primary tumors involving hepatocellular, rectum, and prostate cancers are a rare occurrence with few cases reported in the English literature (Table 1).

During pretreatment work-up of cancer patients, the diagnosis of a second mass raises the possibility either of metastasis or of a synchronous primary malignant tumor. When multiple tumors are pathologically confirmed, the therapeutic approach is decided after staging each tumor independently. Moreover, the patient's age, life expectancy, and comorbidities as well as the tumor's biological behavior should be considered. Treatment options include curative surgical resection of each tumor, when possible in a single setting, radiation, or chemoradiation [26–28]. As there exist no systematic and authoritative treatment guidelines for the treatment of synchronous primary malignancies, the aim is to cover all primaries without negatively affecting the overall patient's outcome.

Especially for triple synchronous carcinomas, treatment strategies remain poorly defined. Several studies have failed to show a significant impact of a second extra hepatic malignancy on the survival of patients with HCC, suggesting that aggressive treatment of HCC by curative resection is an independent prognostic factor associated with a good outcome [29, 30]. In the present case, we prioritized the cure of the hepatocellular carcinoma independently of two other malignancies. However, the successful implementation of hepatectomy represented a therapeutic challenge due to the large size of the tumor and the small functional liver remnant. We opted for sequential TACE and PVE of the hepatocellular carcinoma as this strategy is associated with longer recurrence-free survival [31, 32]. TACE allowed for local control of the hepatocellular carcinoma during which the rectal carcinoma was resected, and PVE was performed allowing eventual radical HCC excision. Taking into account the presence of two other synchronous malignancies and patient's preferences, active therapy of prostate cancer was withheld, and active surveillance was adopted. Recent guidelines for the follow-up of HCC after liver resection recommend dynamic CT/MRI studies every 3 months during the first year and every 6 months thereafter [33]. Follow-up recommendations for rectal carcinoma include rectosigmoidoscopy and CEA testing every 6 months for 5 years and abdominal and chest CT scan annually for 3 years [34]. Our patient kept the above-mentioned follow-up protocol for six months and opted for a simple clinical follow-up for two years, during which he remained in an excellent clinical condition.

## 4. Conclusion

Our case highlights the value of targeted curative therapy of synchronous malignancies in a framework of a patient-centered multidisciplinary approach. We achieved an excellent result with curative therapy of two of the three synchronous lesions and opted for conservative management of the prostate cancer.

## Figures and Tables

**Table 1:** The previously reported examples of synchronous triple primary malignancies including rectal, prostate, and hepatocellular cancers.

Author	Year	Age/gender	Malignancies	Treatment	Follow-up
Okajima et al. [11]	1994	75/M	HCC-RCC-SqCC of the oral floor	Simultaneous resection of all tumors	Not reported
Chang et al. [12]	2003	74/M	HCC-cholangiocarcinoma-GA	Wedge resection of RLL, LL, and subtotal gastrectomy	Under follow-up
Wang et al. [13]	2008	62/M	HCC-basaloid SqCC of the esophagus-SqCC of the gum	Curative surgery for gum and esophagus cancers—no details described about HCC treatment	Died 5 months later of unrelated cause
Lee et al. [14]	2008	69/M	HCC-PC-papillary adenocarcinoma of the gallbladder	Simultaneous wedge resection of the liver segment VII, pylorus-preserving pancreaticoduodenectomy, extended cholecystectomy	Not reported
Lee et al. [15]	2010	56/F	RA-SqCC of cervix-PTC	Uterine conization-neoadjuvant chemoradiation therapy for the RA followed by palliative chemotherapy due to cancer peritonei	12 months
Chong et al. [16]	2010	80/M	HCC-diffuse B-cell gastric lymphoma-GA	No treatment	Died 30 days after diagnosis
Punit Tiwari et al. [17]	2012	55/M	PA-clear cell RCC-bladder TCC	Radical nephroureterectomy followed 3 weeks later by total cystoprostatectomy	2 years of follow-up
Guoliang and Dongsheng [18]	2013	33/M	HCC-CA-partial myxoadenocarcinoma, carcinoid of appendix	Right colectomy-TACE	Died of liver failure 8 months later
Yeh et al. [19]	2013	79/M	RA-cecal adenocarcinoma-right hepatic flexure adenocarcinoma	Simultaneous low anterior resection and an extended right hemicolectomy	Not reported
Ogawa et al. [20]	2014	67/M	A-penile SqCC-SCC of the bladder	Laparoscopic radical cystectomy, urethrectomy, and partial penectomy	Not reported
Cansu et al. [21]	2014	62/M	PA-bladder TCC-PTC	Radical cystectomy followed by chemotherapy-total thyroidectomy 2 months after cystectomy combined with radioactive iodine	Under follow-up
Akiyama et al. [22]	2015	73/M	HCC-CA-SqCC of the esophagus	Simultaneous sigmoidectomy and partial hepatectomy-esophagectomy after 49 days	7 months
Yang et al. [23]	2016	49/M	RA-CA-gastric myxoadenocarcinoma	Simultaneous laparoscopic distal gastrectomy, right hemicolectomy, and radical rectectomy	Not reported
Long et al. [24]	2019	62/M	RA-PA-PTC	Chemoradiotherapy and hormonal therapy for RA and PA-clinical observation of thyroid carcinoma	Stable disease
Mira et al. [25]	2019	71/M	HCC, lung SCC, right tongue SCC	RT for SCC of the lung-partial glossectomy-HCC treatment not reported	One year

Abbreviations: RA: rectal adenocarcinoma; HCC: hepatocellular carcinoma; RCC: renal cell carcinoma; SqCC: squamous cell carcinoma; PTC: papillary thyroid carcinoma; GA: gastric adenocarcinoma; CA: colonic adenocarcinoma; TCC: transitional cell carcinoma; LA: lung adenocarcinoma; SCC: small-cell adenocarcinoma; RT: radiotherapy; LL: left lobectomy; RLL: right liver lobe.
